# Risk factors affecting relapse after discontinuation of biologics in children with Crohn's disease who maintained deep remission

**DOI:** 10.3389/fped.2024.1479619

**Published:** 2024-10-07

**Authors:** Hansol Kim, Yoon Zi Kim, Seon Young Kim, Yon Ho Choe, Mi Jin Kim

**Affiliations:** Department of Pediatrics, Samsung Medical Center, Sungkyunkwan University School of Medicine, Seoul, Republic of Korea

**Keywords:** Crohn’s disease, biologics, relapse, remission, pediatric

## Abstract

**Objectives:**

Biologics are important therapeutic agents for pediatric Crohn's disease. Discontinuation of biologics is known to increase the relapse rate up to 71.4% in these patients; however, their long-term use increases the risk of opportunistic infections and causes economic burden and psychological fatigue. Therefore, taking a drug holiday is meaningful, even if the biologics cannot be completely discontinued. This study aimed to analyze the risk factors affecting relapse after discontinuation of biologics in children with Crohn's disease.

**Methods:**

We retrospectively reviewed the data of 435 children with Crohn's disease who visited a single health center between March 2013 and March 2021. Subsequently, we analyzed data from the patients who discontinued biologics after deep remission.

**Results:**

Among the enrolled patients, 388 were followed up for ≥2 years, and of these, 357 were administered biologics. A total of 103 patients discontinued biologics after deep remission, subsequently 31 maintained remission and 72 relapsed. The shorter the duration of biologic treatment (odds ratio of 0.444, *P* = 0.029), the higher the ESR (odds ratio of 1.294, *P* = 0.009) and fecal calprotectin (odds ratio of 1.010, *P* = 0.032), and the less histological remission at the time of discontinuation of biologics (odds ratio of 0.119, *P* = 0.026), the greater the risk of relapse after discontinuation of biologics.

**Conclusions:**

We identified factors associated with relapse after discontinuation of biologics. The results suggest that biologics can be discontinued in the absence of these factors after deep remission. However, because the relapse rate may increase after the discontinuation of biologics, close monitoring is important, and if necessary, re-administration of biologics should be actively considered.

## 1 Introduction

Crohn's disease (CD) is a chronic inflammatory disease of the gastrointestinal tract characterized by relapsing and remitting symptoms. It is a progressive disease that leads to bowel damage and disability (1). The prevalence of pediatric CD is increasing worldwide (2).

Exclusive enteral nutrition (EEN), corticosteroids, biologics, and immunomodulators are used to treat CD. In particular, biologics are effective in inducing clinical and endoscopic remission (3, 4), and are recommended as primary induction and maintenance therapies for children with a high risk of poor outcomes according to the European Crohn's and Colitis Organization (ECCO)/European Society for Paediatric Gastroenterology Hepatology and Nutrition (ESPGHAN) guidelines (5). Although the top-down strategy of administering biologics in the early and acute stages of the disease is not specified in the recent pediatric guidelines, the strategy to induce rapid remission in children with CD has been associated with favorable outcomes and recent studies have commented this strength (6–8).

However, the relapse rate of CD increases when biologics are discontinued in patients with CD who are in remission (9–11); and factors affecting clinical relapse after infliximab (IFX) or azathioprine (AZA) withdrawal in children with CD on combination therapy have been investigated. IFX cessation increased the relapse risk and 71.4% patients who withdrew IFX experienced relapse (9). Thus, discontinuation of biologics is a difficult decision, and the timing remains controversial (12).

When using biologics, the occurrence of side effects must always be considered; these include opportunistic infections, and malignancies, as well as miscellaneous complications such as reactions to injection or infusion, autoimmune reaction, and cutaneous side effects (13, 14). In addition, the long-term administration of biologics causes an economic burden on patients as well as psychological fatigue owing to continuous and frequent hospital visits and administration of injections. Children are more likely to encounter these problems because the treatment period of disease for children is significantly longer than that for adults. Therefore, the decision to administer biologics throughout life is difficult because of concerns regarding relapse.

Since the possibility of relapse after the discontinuation of biologics is high, taking a drug holiday is important even if biologics cannot be completely discontinued. Discontinuation of biologics could be attempted in patients who can maintain remission for a relatively long period after discontinuing biologics.

This study aimed to analyze factors associated with relapse and the period until relapse after discontinuation of biologics in children with CD who maintained deep remission, and to identify patients who can maintain remission or sustain a long remission and a drug holiday period after discontinuation of biologics.

## 2 Methods

### 2.1 Patients and study design

Data of 435 patients aged <19 years, who visited the Samsung Seoul Hospital between March 2013 and March 2021, and were diagnosed with CD, were retrospectively analyzed. CD was diagnosed according to the Porto criteria of the ESPGHAN (15), was classified according to the Paris classification (16). We divided patients into those who discontinued biologics and continued, regardless of other concurrent non-biologic IBD treatments. These patients discontinued biologics because they sustained deep remission, and their data were analyzed. Although some patients attempted to discontinue biologics more than twice, data were analyzed based on the time point at which biologics were first discontinued after diagnosis.

All the patients underwent periodic blood, stool tests, as well as endoscopy, biopsy, and magnetic resonance enterography (MRE). The outpatient visit schedule varied depending on the period of biologic administration and the patient's condition at intervals of 4–8 weeks. Blood tests were performed at each outpatient visit, and the patient's clinical state was evaluated using the Pediatric Crohn's Disease Activity Index (PCDAI) score (17). In addition, stool calprotectin was requested at one of the two outpatient visits. IFX trough level was checked periodically before every 3–4 biologics discontinuation of biologics is a difficult decision, and the timing remains controversial administrations and additionally when the patients had clinical or biochemical evidence of active disease. Endoscopy was performed along with biopsy of the terminal ileum, cecum, ascending colon, transverse colon, descending colon, sigmoid colon, and rectum. Endoscopy and MRE were performed as follow-up examinations for 1 year after initiating biologic administration and every 2 years thereafter. In patients with confirmed clinical and endoscopic remission, discontinuation of biologics was attempted.

All the methods were performed in accordance with the relevant guidelines and regulations and were approved by the Clinical Research Ethics Committee of the Samsung Medical Center (IRB file no. SMC 2023-05-058-001).

### 2.2 Data collection

Data collected at the time of diagnosis included the patient's sex, age at the time of diagnosis, non-biologic CD treatments administered at the time of diagnosis [steroid, 5-aminosalicylic acid (5-ASA), and immunomodulators]; results of blood tests [hematocrit, albumin, erythrocyte sedimentation rate (ESR), and C-reactive protein (CRP)], clinical symptoms (PCDAI score), endoscopic findings [simple endoscopic score for CD (SES-CD score)], and disease classification (Paris classification).

Data collected at the time of initiating biologics included the type of biologics administered, age at the time of starting biologics, and the duration from diagnosis to biologic administration.

Data collected at the time of biologic discontinuation were based on the time point when the biologics were first discontinued, and included age at the time of discontinuation of biologics, total duration of biologic administration, non-biologic CD treatments (5-ASA or immunomodulators) taken concurrently, blood tests (hematocrit, albumin, ESR, CRP, and IFX trough levels), levels of fecal calprotectin, clinical symptoms (PCDAI score), endoscopic findings (SES-CD score), simplified Magnetic Resonance Index of Activity (MARIA) score, and presence of histological remission. Additionally, the period until relapse after the discontinuation of biologics and total follow-up duration after discontinuation of biologics were recorded.

### 2.3 Definitions

Clinical remission was defined as a PCDAI score of ≤10, and endoscopic remission was defined as a SES-CD score of 0–2. Deep remission was defined by presence of endoscopic remission along with clinical remission. Histological remission was defined as the absence of active inflammation in the terminal ileum, cecum, ascending colon, transverse colon, descending colon, sigmoid colon, and rectum. Relapse was defined as clinical relapse (PCDAI score >10) and/or exhibiting increased in inflammatory marker levels (ESR, CRP, and fecal calprotectin) after discontinuation of biologics, and addition of a new medicine or escalation of maintenance therapy.

### 2.4 Statistical analyses

Categorical variables are expressed as percentages, and continuous variables are expressed as medians and interquartile ranges (IQRs). The Mann–Whitney *U*-test was performed to compare continuous variables and the chi-square and Fisher's exact tests were performed to compare categorical variables between the remission and relapse group. Univariate and multivariable logistic regression analyses were performed to find risk factors that affected the relapse after discontinuation of biologics. The chi-square and Fisher's exact tests were performed to compare categorical variables between the group that relapsed within 12 months after the discontinuation of biologics and the group that relapsed after ≥12 months. The period from discontinuation of biologics until relapse appeared to a skewed distribution; therefore, log transformation was performed. After confirming that it was a normal distribution, a Pearson's correlation analysis was conducted to find the correlation between the period from discontinuation of biologics until relapse and continuous variables. All statistical analyses were performed using the IBM SPSS Statistics for Windows version 28. *P*-value <0.05 was considered statistically significant.

## 3 Results

### 3.1 Clinical characteristics and comparison between patients who maintained remission and relapsed after discontinuation of biologics

**Figure 1:**
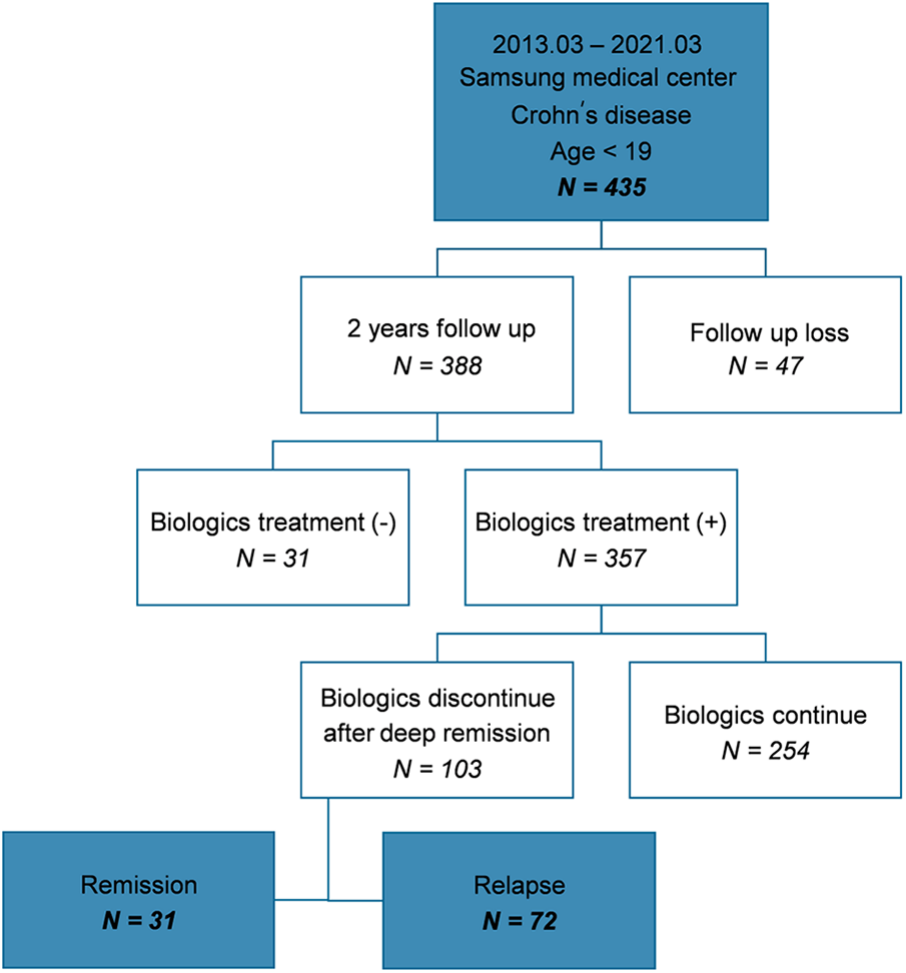
Flow chart showing the patient selection process and patient characteristics.

Among the 435 patients included in the study, 388 were followed-up for ≥2 years at the hospital; of these, 357 patients with moderate-to-severe disease were administered biologics. In total, 103 patients attempted to discontinue biologics because they sustained deep remission. Subsequently, 31 patients maintained remission (group A) and 72 patients relapsed (group B) (Figure 1).

The clinical characteristics and comparison between group A and B after discontinuation of biologics were analyzed (Table 1). The average period of relapse after discontinuation of biologics was 19.07 months, the median was 14.5 months, the IQR was 8–24 months, the minimum was 1 month, and the maximum was 80 months. We observed that 32 patients (44%) relapsed within 12 months, although some patients maintained remission for up to 80 months. Thus, the period from discontinuation of biologics until relapse varied for each patient. The median of follow-up duration after discontinuation of biologics was 31 months in group A, and 41.5 months in group B. There was no statistically significant difference in total follow-up duration between two groups (*P* = 0.077).

**Table 1:** Clinical characteristics and comparison between patients who maintained remission and relapsed after discontinuation of biologics.

	Remission (*n* = 31)	Relapse (*n* = 72)	*P*-value
At diagnosis			
Sex, *n* (%)			
Male	18 (58.1%)	53 (73.6%)	0.118^b^
Female	13 (41.9%)	19 (26.4%)	
Age at diagnosis, years (IQR)	15.0 (11.0–17.0)	14.0 (12.0–15.0)	0.284^a^
Medication, *n* (%)			
Steroid	6 (19.4%)	11 (15.3%)	0.609^b^
5-ASA	27 (87.1%)	68 (94.4%)	0.238^c^
Immunomodulator	26 (83.9%)	53 (73.6%)	0.259^b^
Laboratory findings (IQR)			
Hematocrit, %	37.3 (33.3–39.9)	35.9 (32.8–39.8)	0.428^a^
Albumin, g/dl	3.9 (3.4–4.4)	3.8 (3.3–4.1)	0.211^a^
ESR, mm/h	51 (27–71.2)	60 (26.5–74.5)	0.540^a^
CRP, mg/dl	3.43 (0.3–7.96)	2.49 (0.95–5.71)	0.981^a^
PCDAI score (IQR)	37.5 (30.0–44.3)	32.5 (20–45)	0.244^a^
SES-CD score (IQR)	15 (9–21)	16 (11–21)	0.736^a^
CD age, *n* (%)			
A1a (0–<10 years)	5 (16.1%)	3 (4.2%)	0.067^b^
A1b (10–<17 years)	18 (58.1%)	60 (83.3%)	
A2 (17–40 years)	8 (25.8%)	9 (12.5%)	
CD location, *n* (%)			
L1 (ileal)	2 (6.5%)	2 (2.8%)	0.582^c^
L2 (colonic)	4 (12.9%)	5 (6.9%)	0.447^c^
L3 (ileocolonic)	24 (77.4%)	64 (88.9%)	0.221^c^
L4a (proximal upper disease)	7 (22.6%)	20 (27.8%)	0.582^b^
L4b (distal upper disease)	5 (16.1%)	16 (22.2%)	0.481^b^
CD behavior, *n* (%)			
B1 (nonstricturing, nonpenetrating)	26 (83.9%)	60 (83.3%)	0.946^b^
B2 (stricturing)	5 (16.1%)	12 (16.7%)	
B3 (penetrating)	0 (0%)	0 (0%)	
Perianal disease, *n* (%)	19 (61.3%)	56 (77.8%)	0.085^b^
CD growth, *n* (%)			
G0 (no evidence of growth delay)	28 (96.6%)	51 (85.0%)	0.157^c^
G1 (growth delay)	1 (3.4%)	9 (15.0%)	
At the start of biologic use			
Type of biologics, *n* (%)			
Infliximab	24 (77.4%)	53 (73.6%)	0.683^b^
Adalimumab	7 (22.6%)	19 (26.4%)	
Period from diagnosis to biologic administration, months (IQR)	2.0 (0.0–26.0)	2.0 (0–11.2)	0.936^a^
Age at the start of biologic use, years (IQR)	16.0 (13.0–18.0)	15.0 (13.0–17.0)	0.167^a^
At discontinuation of biologics			
Total follow-up duration after discontinuation of biologics, months (IQR)	31.0 (15.5–56.5)	41.5 (32.0–58.0)	0.077^a^
Duration of biologic treatment, years (IQR)	3.0 (1.6–5.5)	2.0 (1.0–2.5)	**0.001** ^a^
Age at discontinuation of biologics, years (IQR)	19.0 (17.0–22.0)	16.0 (15.0–19.0)	**0.005** ^a^
Medication, *n* (%)			
Yes	16 (51.6%)	53 (73.6%)	**0** **.** **029** ^b^
None	15 (48.4%)	19 (26.4%)	
Laboratory findings (IQR)			
Hematocrit, %	42.3 (39.3–46.7)	42.1 (38.2–44.8)	0.470^a^
Albumin, g/dl	4.6 (4.4–4.7)	4.5 (4.3–4.7)	0.628^a^
ESR, mm/h	4 (2–7)	8 (4–16.7)	**0** **.** **004** ^a^
CRP, mg/dl	0.06 (0.04–0.06)	0.04 (0.03–0.20)	0.594^a^
Infliximab trough level, mcg/ml	3.8 (0.9–8.3)	3.8 (1.9–6.5)	0.898^a^
Fecal calprotectin, mg/kg (IQR)	21.0 (11.2–39.6)	178.5 (39.1–489.1)	**<0.001** ^a^
PCDAI score (IQR)	0 (0–0)	0 (0–5)	**0** **.** **037** ^a^
SES-CD score (IQR)	0 (0–1)	0 (0–1)	0.891^a^
Simplified MARIA score (IQR)	0 (0–1)	1 (1–1)	**<0** **.** **001** ^a^
Histological remission, *n* (%)			
Yes	29 (93.5%)	33 (46.5%)	**<0** **.** **001** ^b^
No	2 (6.5%)	38 (53.5%)	

IQR, interquartile range; 5-ASA, 5-aminosalicylic acid; ESR, erythrocyte sedimentation rate; CRP, C-reactive protein; PCDAI, pediatric Crohn's disease activity index; SES-CD, simple endoscopic score for Crohn's disease; CD, Crohn's disease.

Bold values mean statistically significant values with *p*-value < 0.05.

^a^ Mann-Whitney *U*-test.

^b^ Chi-squared test.

^c^ Fisher's exact test.

At the time of diagnosis, the group who would stay in remission following discontinuation of biologics comprised 18 boys (58.1%) and 13 girls (41.9%) and the group who would relapse following discontinuation of biologics comprised 53 boys (73.6%) and 19 girls (26.4%), and the median age at diagnosis was 15.0 and 14.0 years. Non-biologic CD treatments that were initiated at the time of diagnosis included steroids, 5-ASA, and immunomodulators, which were identified in 6 (19.4%), 27 (87.1%), and 26 (83.9%) in group A and 11 (15.3%), 68 (94.4%), and 53 (73.6%) patients in group B, respectively; 5-ASA was administered to the largest number of patients. The median values of laboratory findings at the time of diagnosis were as follows; hematocrit, 37.3%; albumin, 3.9 g/dl; ESR, 51 mm/h; CRP, 3.43 mg/dl in group A and hematocrit, 35.9%; albumin, 3.8 g/dl; ESR, 60 mm/h; and CRP, 2.49 mg/dl in group B. The median values of the PCDAI and SES-CD scores were 37.5 and 15 in group A and 32.5 and 16 in group B, respectively. According to the Paris classification, most patients were A1b, L3, and B1 in both groups; 19 (61.3%) patients had perianal disease in group A and 56 (77.8%) patients had perianal disease in group B, and 1 (3.4%) had a growth delay in group A and 9 (15.0%) had a growth delay in group B.

The biologics administered included IFX in 24 (77.4%) and adalimumab (ADL) in 7 (22.6%) patients in group A and IFX in 53 (73.6%) and ADL in 19 (26.4%) patients in group B. The median period from diagnosis to administration of biologics was generally short at 2 months in both groups. The median age at which biologic administration was initiated was 16.0 and 15.0 years in each groups. A statistically significant difference in the median value of the total period of biologic administration was observed between the two groups (*P* = 0.001).

The median value of total period of biologic administration was 3.0 years in group A and 2.0 years in group B. A statistically significant difference in the median age at the time of discontinuation of biologics was observed between the two groups (*P* = 0.005). The median age at the time of discontinuation of biologics was 19.0 years in group A and 16.0 years in group B. At the time of biologic discontinuation, 15 (48.4%) patients were not taking non-biologic CD treatments in group A and 19 (26.4%) patients were not taking non-biologic CD treatments in group B and statistically significant difference was observed (*P* = 0.029). At the time of discontinuation of biologics, the median values of laboratory findings were as follows; hematocrit, 42.3%; albumin, 4.6 g/dl; ESR, 4 mm/h; CRP, 0.06 mg/dl; and IFX trough level, 3.8 mcg/mL in group A and hematocrit, 42.1%; albumin, 4.5 g/dl; ESR, 8 mm/h; CRP, 0.04 mg/dl; and IFX trough level, 3.8 mcg/ml in group B. The median value of fecal calprotectin, PCDAI and SES-CD scores, and the simplified MARIA score were 21.0 mg/kg, 0, 0, and 0, respectively in group A and 178.5 mg/kg, 0, 0, and 1, respectively in group B. Histological remission was confirmed in 29 (93.5%) patients in group A and 33 (55.9%) patients in group B. A statistically significant difference in the ESR, fecal calprotectin, PCDAI, simplified MARIA score and histological remission at the time of discontinuation of biologics were observed between the two groups, respectively (*P* = 0.004, <0.001, 0.037, <0.001 and <0.001).

### 3.2 Logistic regression analysis

A logistic regression analysis was performed to identify the risk factors that affected the relapse after discontinuation of biologics (Table 2). Variables with statistically significant differences between group A and B were included and analyzed; duration of biologics treatment, age, presence of non-biologic CD treatments taken concurrently, ESR, fecal calprotectin, PCDAI score, simplified MARIA score, and presence of histological remission at the time of discontinuation of biologics. First, the univariate analysis was performed, and variables with a confirmed *p*-value of <0.1 were selected and then, a multivariable analysis was performed. In multivariable analysis, the shorter the duration of biologic treatment (odds ratio of 0.444, *P* = 0.029), the higher the ESR (odds ratio of 1.294, *P* = 0.009) and fecal calprotectin (odds ratio of 1.010, *P* = 0.032), and the less histological remission at the time of discontinuation of biologics (odds ratio of 0.119, *P* = 0.026), the greater the risk of relapse after discontinuation of biologics.

**Table 2:** Factors affecting the relapse after discontinuation of biologics.

	Univariate analysis				Multivariable analysis			
	*P*-value	Odds ratio	95% CI		*p*-value	Odds ratio	95% CI	
			Lower	Upper			Lower	Upper
Duration of biologic treatment	**<0.001**	**0** **.** **602**	0.456	0.796	**0** **.** **029**	**0** **.** **444**	0.214	0.919
Age at d/c of biologics	**0.007**	**0.846**	0.749	0.956				
Medication (yes or no) at d/c of biologics	**0.032**	**0.382**	0.159	0.920				
ESR at d/c of biologics	**0.014**	**1.099**	1.019	1.186	**0.009**	**1.294**	1.067	1.569
Fecal calprotectin at d/c of biologics	**0.003**	**1.010**	1.004	1.017	**0.032**	**1.010**	1.001	1.020
PCDAI at d/c of biologics	0.250	1.067	0.955	1.191				
Simplified MARIA score at d/c of biologics	**<0.001**	**4.810**	2.030	11.397				
Histological remission at d/c of biologics	**<0.001**	**0.060**	0.013	0.270	**0.026**	**0.119**	0.018	0.776

The risk factors associated with the relapse after discontinuation of biologics were identified using a logistic regression analysis model.

ESR, erythrocyte sedimentation rate; PCDAI, pediatric Crohn's disease activity index; MARIA, magnetic resonance index of activity.

Bold values mean statistically significant values with *p*-value < 0.1 in univariate analysis and < 0.05 in multivariable analysis.

### 3.3 Correlation analysis

**Figure 2:**
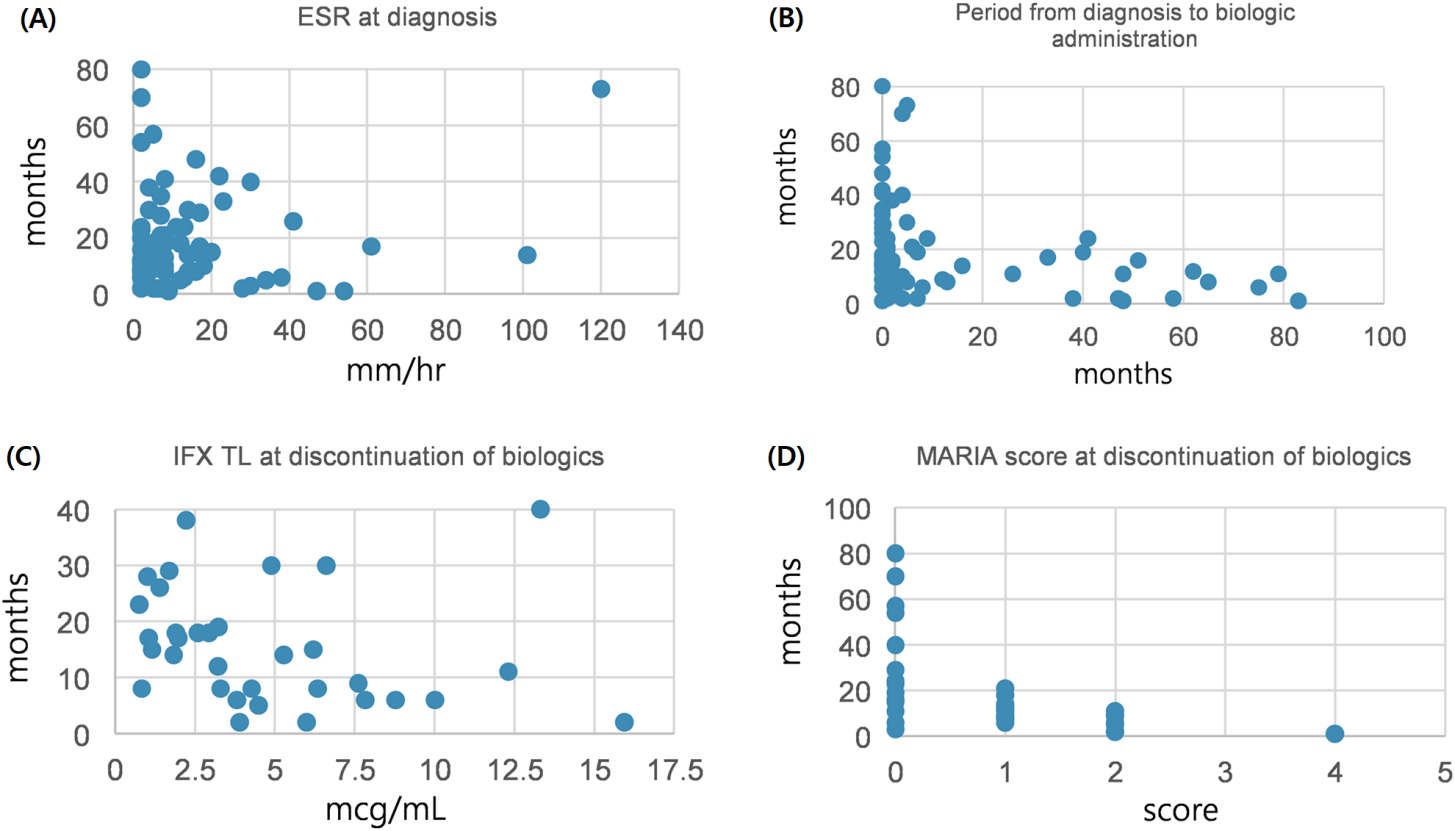
Correlation between continuous variables and period until relapse after discontinuation of biologics. **(A)** Between ESR at diagnosis and period until relapse after discontinuation of biologics. **(B)** Between period diagnosis to biologic administration and period until relapse after discontinuation of biologics. **(C)** Between IFX TL at discontinuation of biologics and period until relapse after discontinuation of biologics. **(D)** Between MARIA score at discontinuation of biologics and period until relapse after discontinuation of biologics. ESR, erythrocyte sedimentation rate; IFX TL, infliximab trough level; MARIA, magnetic resonance Index of activity.

After comparison between group A and B, we analyzed the variables correlated with the period from discontinuation of biologics until relapse. Patients relapsed after discontinuation of biologics were classified into two groups based on the period from discontinuation of biologics until relapse (within 12 months or ≥12 months). We investigated any significant differences between categorical variables in these two groups, and no significant differences in all the variables were observed (Table 3). A correlation analysis was performed to confirm the relationship between the period from discontinuation of biologics until relapse and the continuous variables. The period from discontinuation of biologics until relapse appeared a skewed distribution; therefore, log transformation was performed. After confirming a normal distribution, a Pearson's correlation analysis was performed (Table 4). Subsequently, statistically significant associations were confirmed for the four variables. The ESR at the time of diagnosis (*r* = –0.304, *P* = 0.017), period from diagnosis to biologic administration (*r* = –0.384, *P* < 0.001), IFX trough level (*r* = –0.368, *P* = 0.035), and a simplified MARIA score at the time of discontinuation of biologics (*r* = –0.730, *P* < 0.001) showed a negative correlation with the period from discontinuation of biologics until relapse (Figure 2).

**Table 3:** Comparison of categorical variables between two groups (patients who relapsed within 12 months after discontinuation of biologics vs. patients who relapsed ≥12 months after discontinuation of biologics).

	≤12 months (*n* = 32)	>12 months (*n* = 40)	*P*-value
At diagnosis			
Sex, *n* (%)			
Male	24 (75.0%)	29 (72.5%)	0.811^a^
Female	8 (25.0%)	11 (27.5%)	
Medication, *n* (%)			
Steroid	3 (9.4%)	8 (20.0%)	0.325^a^
5-ASA	32 (100.0%)	36 (90.0%)	0.124^a^
Immunomodulator	23 (71.9%)	30 (75.0%)	0.765^a^
CD age, *n* (%)			
A1a (0–<10 years)	0 (0%)	3 (7.5%)	0.414^b^
A1b (10–<17 years)	28 (87.5%)	32 (80.0%)	
A2 (17–40 years)	4 (12.5%)	5 (12.5%)	
CD location, *n* (%)			
L1 (ileal)	1 (3.1%)	1 (2.5%)	1.000^b^
L2 (colonic)	2 (6.3%)	3 (7.5%)	1.000^b^
L3 (ileocolonic)	28 (87.5%)	36 (90.0%)	1.000^b^
L4a (proximal upper disease)	10 (31.3%)	10 (25.0%)	0.556^a^
L4b (distal upper disease)	8 (25.0%)	8 (20.0%)	0.612^a^
CD behavior, *n* (%)			
B1 (nonstricturing, nonpenetrating)	27 (84.4%)	33 (82.5%)	0.832^a^
B2 (stricturing)	5 (15.6%)	7 (17.5%)	
B3 (penetrating)	0 (0%)	0 (0%)	
Perianal disease, *n* (%)			
Yes	23 (71.9%)	33 (82.5%)	0.281^a^
No	9 (28.1%)	7 (17.5%)	
CD growth, *n* (%)			
G0 (no evidence of growth delay)	26 (89.7%)	25 (80.6%)	0.474^b^
G1 (growth delay)	3 (10.3%)	6 (19.4%)	
At start of biologic use			
Type of biologics, *n* (%)			
Infliximab	22 (68.8%)	31 (77.5%)	0.402^a^
Adalimumab	10 (31.2%)	9 (22.5%)	
At discontinuation of biologics			
Medication, *n* (%)			
Yes	26 (81.2%)	27 (67.5%)	0.188^a^
None	6 (18.8%)	13 (32.5%)	
Histological remission, *n* (%)			
Yes	15 (46.9%)	11 (27.5%)	0.291^b^
No	13 (40.6%)	20 (50.0%)	

5-ASA, 5-aminosalicylic acid; CD, Crohn's disease.

^a^ Chi-squared test.

^b^ Fisher's exact test.

**Table 4:** Pearson's correlation analysis between period until relapse after discontinuation of biologics and continuous variables.

	Pearson's coefficient of correlation	*P*-value
At diagnosis		
Age at diagnosis, years (IQR)	0.007	0.954
Laboratory findings (IQR)		
Hematocrit, %	0.084	0.521
Albumin, g/dl	0.092	0.479
ESR, mm/h	**−0.304**	**0** **.** **017**
CRP, mg/dl	−0.049	0.701
PCDAI score (IQR)	0.074	0.577
SES-CD score (IQR)	0.047	0.702
At start of biologics		
Period from diagnosis to biologic administration, months (IQR)	**−0** **.** **384**	**<0** **.** **001**
Age at start of biologics, years (IQR)	−0.229	0.053
At discontinuation of biologics		
Duration of biologic treatment, years (IQR)	0.017	0.889
Age at discontinuation of biologics, years (IQR)	−0.204	0.086
Laboratory findings (IQR)		
Hematocrit, %	−0.063	0.599
Albumin, g/dl	−0.082	0.491
ESR, mm/h	−0.007	0.952
CRP, mg/dl	0.082	0.492
Infliximab trough level, mcg/ml	**−0.368**	**0** **.** **035**
Fecal calprotectin, mg/kg (IQR)	0.033	0.874
PCDAI score (IQR)	−0.076	0.629
SES-CD score (IQR)	−0.170	0.199
Simplified MARIA score (IQR)	**−0.730**	**<0.001**

IQR, interquartile range; ESR, erythrocyte sedimentation rate; CRP, C-reactive protein; PCDAI, pediatric Crohn's disease activity index; SES-CD, simple endoscopic score for Crohn's disease; MARIA, magnetic resonance index of activity.

Bold values mean statistically significant values with *p*-value < 0.05.

### 3.4 Comparison between patients who were taking non-biologic CD treatments at discontinuation with those who were not

69 (67.0%) patients were taking non-biologic CD treatments and 34 (33.0%) patients were not taking at the time of discontinuation of biologics. A statistically significant difference in the concurrently non-biologic CD treatments at the time of discontinuation of biologics was observed between group A and B. Therefore, we compared these two groups and analyzed the correlation with other variables at the time of discontinuation of biologics. Hematocrit, albumin, ESR, CRP, fecal calprotectin, PCDAI score, SES-CD score and simplified MARIA score in the patients who were taking non-biologic CD treatments at the time of discontinuation of biologics were as follows; 41.2%, 4.5 g/dl, 8.0 mm/h, 0.04 mg/dl, 131.35 mg/kg, 0, 0, and 1. The same laboratory findings in the patients who were not taking non-biologic CD treatments at the time of discontinuation of biologics were as follows; 43.5%, 4.5 g/dl, 6.0 mm/h, 0.04 mg/dl, 32.5 mg/kg, 0, 0, and 0. In the group who were taking non-biologic CD treatments at the time of discontinuation of biologics, 34 (50.0%) patients were maintaining histological remission and in those who were not, 28 (82.4%) patients were maintaining histological remission. A statistically significant difference in the hematocrit and histological remission at the time of discontinuation of biologics was observed between the two groups, respectively (*P* = 0.038 and 0.002).

### 3.5 Clinical course and antibody formation of patients who relapsed after discontinuation of biologics

**Figure 3:**
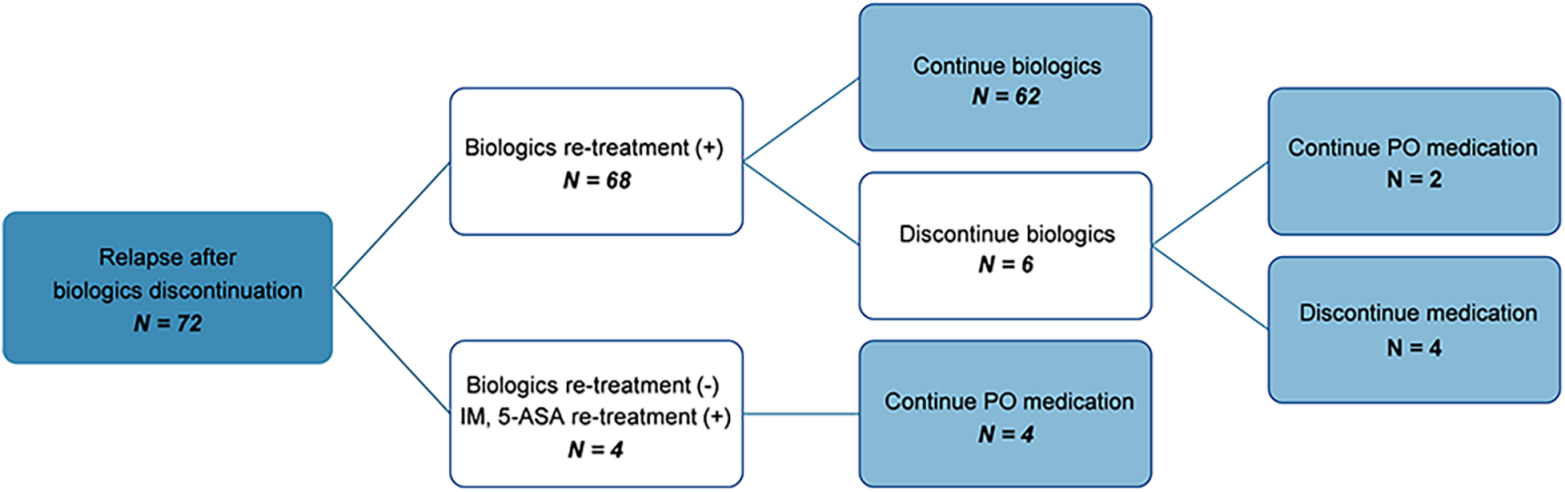
Flow chart showing the clinical course of patients who relapsed after discontinuation of biologics. IM, immunomodulators; 5-ASA, 5-Aminosalicylate; PO, *per os*.

The clinical course of patients who experienced relapse after discontinuation of biologics was as follows (Figure 3): Among the 103 patients who attempted to discontinue biologics, 31 maintained remission, whereas 72 relapsed. Among those who relapsed, 68 were re-administered biologics, of whom 62 successfully maintained remission. When re-administering biologics, all patients were treated with the same biologics they had taken before discontinuing the biologics. Of these, only one patient changed biologics from IFX to ADL because of infusion reaction. 67 patients (98.5%) maintained remission without infusion reaction after re-administration of biologics. Subsequently, six patients made a second attempt at discontinuation, with two maintaining remission on non-biologic CD treatments and four without any medication.

Additionally, no severe complications, such as strictures or the need for colectomy, were reported during relapse after the initial discontinuation of biologics. However, 3 patients developed complications, including strictures and colectomies for long-term follow-up duration (maximum 8.8 years). One patient underwent balloon dilatation because of terminal ileum stricture during biologics re-administration after relapse. One underwent small bowel resection & anastomosis because of distal ileum perforation 3 years after discontinuing biologics and the compliance was poor as the patient did not take non-biologic CD treatment. One failed to be followed up for 2 years after discontinuing biologics and subsequently underwent descending colon resection & anastomosis because of colo-colonic intussusception.

Data analyses in the present study showed that of the 52 patients who underwent continuous antibody testing, 49 consistently tested negative, while two tested positive even before discontinuation. One patient (1.9%) experienced an infusion reaction during the re-administration of biologics after discontinuation, leading to a change in biologics from IFX to ADL.

## 4 Discussion

This study retrospectively analyzed patient data over an 8-year period to identify factors associated with relapse and the period from discontinuation of biologics to relapse. Biologics are crucial for maintaining remission (3, 4); however, relapse rates often increase after their discontinuation (9–11). Discontinuing the biologic drug IFX increased the relapse risk in children, whereas discontinuing AZA did not (9). However, because the medication cannot be administered for a lifetime, a drug holiday period is necessary, and the timing of discontinuation of biologics needs to be considered carefully. Presence of relapse and the time until relapse after the discontinuation of biologics varies among patients. Therefore, we believed that patients who can maintain remission after discontinuation of biologics could attempt to discontinue the drug. Also the patients who maintain a long remission period from the discontinuation of biologics until relapse could attempt to discontinue the drug even if biologics are administered again after relapse. Many studies have investigated factors affecting relapse after discontinuation of biologics in patients with inflammatory bowel diseases and suggested stopping biologics and taking a drug holiday (18–21).

This study aimed to compare the differences between patients who stayed in remission following discontinuation of biologics and relapsed and analyze the risk factors associated with the relapse after discontinuation of biologics in children with CD who maintained deep remission. And we analyzed variables correlated with the relapse period after discontinuation of biologics, determine which patients could have a long remission period and drug holiday period after discontinuation of biologics, and provide suggestions on discontinuation of biologics in these patients.

We obtained several important findings from this study. The median duration of biologics treatment was 3.0 years in group A, and 2.0 years in group B, indicating that the duration of biologics treatment is a risk factor affecting the relapse after discontinuation of biologics. Biologics such as infliximab and adalimumab inhibit TNF-α, which is a cytokine involved in normal inflammatory and immunological responses (22) so discontinuation of biologics when bowel inflammation was sufficiently treated is important in maintaining subsequent remission. However, there was no statistically significant difference in the type of biologics (*P* = 0.683). Duration of biologics treatment is important regardless of the type of biologics to maintain remission after discontinuation of biologics. We found that the group maintained remission had a higher age at the time of discontinuation of biologics, which can be inferred to be a result of the longer period of biologics treatment.

We observed the differences in non-biologic CD treatments at the time of discontinuation of biologics between two groups and more patients in group A stopped taking non-biologic CD treatments at the time of discontinuation of biologics compared to group B. In analyzing these two groups, those who were not taking non-biologic CD treatments had higher hematocrit levels than those who were taking them, and a higher proportion indicated histological remission at biologic discontinuation. Although the hematocrit levels were within the normal range for both groups, the difference in histological remission indirectly suggests that patients achieving histological remission at biologic discontinuation tend to maintain remission. This implies that maintaining deep remission and effective disease control without non-biologic CD treatments may lead to maintain remission after biologic discontinuation. Additionally, some studies have suggested a lack of association between the AZA discontinuation and CD recurrence. AZA cessation was not associated with clinical relapse, and withdrawal of AZA was considered in children with CD who had sustained clinical remission for at least 2 years and achieved deep remission (9). Thus, maintaining deep and histological remission without non-biologic CD treatments may justify the discontinuation of biologics.

ESR, fecal calprotectin, PCDAI score, and simplified MARIA score are indicators of the activity of CD and of these, ESR and fecal calprotectin at the time of discontinuation of biologics were identified as risk factors affecting relapse after discontinuation of biologics. ESR is a recognized biomarker of CD (23) and correlates with disease activity in patients with inflammatory bowel disease (24–26). It is part of the PCDAI score reflecting disease severity in children (17). Furthermore, the ESR reflects chronic or subacute inflammation, indicating a slower response to inflammation compared to CRP, which increases in response to the acute-phase reaction (27, 28). In particular, higher ESR levels at the time of discontinuation of biologics, reflecting greater disease activity and chronic inflammation, are associated with relapse after discontinuation of biologics.

Fecal calprotectin has adequate sensitivity and specificity to distinguish inflammatory bowel disease from other bowel diseases and we can recognize disease activity and response to treatment by testing fecal calprotectin easily because it is non-invasive test. And an increase in fecal calprotectin can predict an imminent clinical relapse of IBD allowing prompt initiation of treatment (29). We found that fecal calprotectin, which directly reflect bowel inflammation is a risk factor affecting relapse after discontinuation of biologics and confirming the normalization of fecal calprotectin before attempting to discontinue biologics is necessary to maintain the remission.

Histological remission in either UC or CD is currently not considered a clinical target. However, histological remission is likely to be of greater value for improving patient outcomes and reducing disease related complications (30). Patients who achieved histological remission had a lower risk of treatment failure compared with patients with persistent histological activity and these findings support examining histological remission as a potential treatment endpoint in patients with CD (31). We also found that histological remission could affect the relapse after discontinuation of biologics. Determining histological remission as the final treatment target and we can attempt to discontinue biologics.

Additionally, we analyzed group B separately and found the factors associated with the period from discontinuation of biologics to relapse. Low ESR at diagnosis, short period from diagnosis to biologics treatment, low IFX TL and simplified MARIA score at the time of discontinuation of biologics were associated with the period from discontinuation of biologics to relapse. This study revealed that maintaining a longer period of remission after discontinuation of biologics was associated with lower ESR at the time of diagnosis. ESR at the time of discontinuation of biologics was identified as a risk factor for relapse, whereas ESR at the time of diagnosis was associated with the period from discontinuation of biologics to relapse. We found that ESR was a factor associated with both the presence of relapse and the period until relapse after discontinuation of biologics.

**Figure 4:**
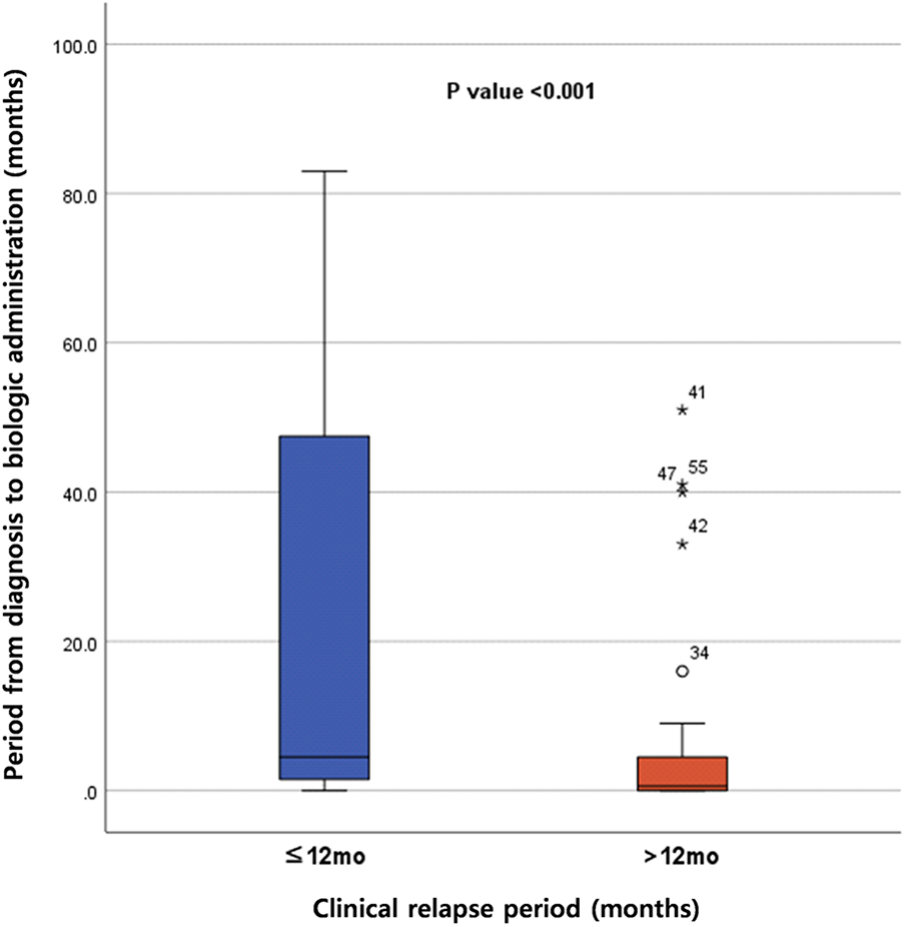
Comparison of period from diagnosis to administration of biologics between two groups (patients who relapsed within 12 months after discontinuation of biologics vs. patients who relapsed >12 months after discontinuation of biologics).

Administering biologics early after diagnosis was associated with a longer duration until relapse after discontinuation of biologics, leading to the maintenance of a longer period of remission. Many previous studies have reported the top-down approach of initiating biologics early after diagnosis, showing improved outcomes compared with the step-up strategy. Walters et al. have reported higher corticosteroid and surgery-free remission rates at 1 year in the biologic group than in the immunomodulatory group with EEN or corticosteroids (6). Kugathasan et al. have reported a reduced risk of complications with biologic treatment (32). Further, mucosal healing rates are higher and remission maintenance is longer with the top-down strategy than with the step-up strategy (7, 8). Early biologic use in both adult and children with CD has demonstrated better clinical remission, reduced relapse rates, and improved mucosal healing than late conventional treatment (33). Studies comparing top-down and step-up treatments with ADL have demonstrated superior maintenance of remission with the top-down therapy (34). The present study also indicates that the top-down strategy is associated with delayed relapse after discontinuation of biologics, which is consistent with previous research and the ECCO/ESPGHAN guidelines advocating early biologic use (5). Additionally, we conducted a minimum *p*-value approach using logistic regression analysis to focus on the impact of the period from diagnosis to the initiation of biologic therapy on the period until relapse after discontinuation. A minimum *p*-value of 0.0066 for the 12-month relapse period indicated the strongest correlation between these variables. Comparison of patients who relapsed within 12 months after discontinuation with those who relapsed beyond 12 months revealed statistically significant differences in the time from diagnosis to biologic initiation, with patients who relapsed earlier having a longer interval (*P* < 0.001) (Figure 4).

Low IFX trough level at the time of discontinuation of biologics was associated with a longer duration until relapse after discontinuation of biologics. This finding aligns with that of previous research, indicating a link between relapse and IFX trough levels at the time of discontinuation of biologics. Because the patients maintained remission despite of low IFX trough levels, their disease may not be considered significantly affected by IFX. IFX trough level more than 2.5 mcg/ml at IFX cessation was positively associated with clinical relapse and IFX trough level at IFX cessation was the only factor associated with clinical relapse in children with CD (35). Louis et al. have reported that high IFX trough levels were associated with relapse after the discontinuation of biologics (36). Papamichael et al. have reported that IFX trough concentrations lower than 6 mcg/ml at the time of IFX discontinuation were associated with sustained clinical remission (37), and Ben-Horin et al. have reported that undetectable anti-tumor necrosis factor (TNF) drug levels were associated with clinical remission (38).

Low simplified MARIA score at the time of discontinuation of biologics was associated with a longer duration until relapse after discontinuation of biologics. Thus, if mucosal healing, confirmed by MRE, is achieved at the time of biologic discontinuation, remission could be sustained for an extended period afterward. MRE has been validated as a non-invasive imaging biomarker of CD mucosal healing (39), rendering it important to actively conduct MRE to assess mucosal healing, particularly for the small bowel. This assessment could be considered a criterion for discontinuing biologics.

The clinical course of patients who experienced relapse after discontinuation of biologics was diverse. As a result, of the 72 patients who discontinued biologics, 62 re-administered and maintained biologics after relapse, and 10 maintained remission without biologics. Therefore, 41 patients (39.8%) succeeded in discontinuing biologics. During the long-term follow up period, 3 patients developed complications, including strictures and colectomies; 2 patients had poor compliance and they were in cases with loss to follow-up and without close monitoring for disease aggravation. Close monitoring after discontinuation of biologics is crucial for promptly detecting disease aggravation and establishing appropriate treatment strategies, such as re-administering biologics, to prevent such complications. However, the discontinuation of biologics cannot be regarded as the only risk factor for stricture or colectomy, considering that the probability of requiring surgery increases with prolonged duration of CD diagnosis (40, 41). Moreover, discontinuation of biologics leads to an increased risk of antibody formation and infusion reactions (42). In this study, one patient (1.9%) experienced an infusion reaction during the re-administration of biologics after discontinuation, leading to a change in biologics from IFX to ADL. However, antibody formation was observed in approximately 28.0% of the patients receiving IFX monotherapy and 7.5% of the patients receiving ADL monotherapy (43). Therefore, considering discontinuation of biologics as the only factor affecting antibody formation is challenging. Nevertheless, discontinuation of biologics may increase the risk of antibody formation, and close monitoring of biologic trough levels and antibody formation is necessary during re-administration to effectively mitigate these risks (42).

Furthermore, the safety of re-administering biologics after relapse has been established in other studies. Molander et al. have reported that restarting TNF1 antagonists elicited an effective and well-tolerated response (44), while Casanova et al. have reported that retreatment with the same anti-TNF drug after relapse was both effective and safe (45). Consequently, if a relapse occurs following the discontinuation of biologics, re-administration of biologics can be actively considered as a safe option.

This study has some limitations. First, it has a retrospective design and was conducted at a single tertiary center, which may have resulted in variations in data collection time points. Data at the time of diagnosis might be missing for patients diagnosed elsewhere and later referred to the institution. However, we tried to analyze a lot of variables to find the risk factors that affect relapse. Second, the risk factors associated with relapse were analyzed at the time the data were collected and the risk of relapse increases over time after discontinuation of biologics in patients who are currently on remission. Therefore, long-term monitoring of whether these patients consistently maintain remission is required and further analysis and research are needed. Third, several patients' age were over 19 years at the time of discontinuation of biologics because it took some months to years to treat biologics. However, the patients enrolled this study were diagnosed at the age <19 years, therefore this is a study of patients who were diagnosed in childhood and adolescence. Nevertheless, the strength of this study is that it was conducted at a center in Korea that treats the largest number of children with CD. Also, this center is actively pursuing a top-down strategy and has extensive experience in using biologics as well as discontinuing biologics after deep remission.

Regular and close monitoring is essential in CD because of the high risk of relapse after discontinuation of biologics. Fecal calprotectin and CRP levels predict relapse (46–49). Monitoring allows for timely intervention, such as restarting or dose intensification of non-biologic CD treatments or restarting biologics, and prevents discomfort. Moreover, when attempting to discontinue biologics, providing sufficient warnings and explanations to patients and their caregivers regarding the risk of relapse is crucial. Discontinuation should be considered if the patient fully understands and agrees with this risk. In clinical practice, many patients and caregivers express concerns regarding the economic burden and psychological fatigue associated with long-term biologic use. Therefore, considering the findings of this study, a drug holiday or discontinuation of biologics should be considered, depending on individual circumstances and patient references.

In conclusion, this study aimed to identify the factors associated with relapse and the period between discontinuation of biologics and relapse in children with CD. The key findings showed that factors such as duration of biologics treatment, ESR, fecal calprotectin and histological remission at the time of discontinuation of biologics were risk factors affecting the relapse after discontinuation of biologics. Considering these factors, cautious discontinuation of biologics may be considered for patients expected to maintain remission after discontinuation of biologics. Moreover, close monitoring and active re-administration of biologics are crucial because of the increased relapse risk.

## Data Availability

The original contributions presented in the study are included in the article/Supplementary Material, further inquiries can be directed to the corresponding author.
